# Investigating Country-Specific Perceptions of Predatory Journals and Their Impact on Scholarly Integrity: A Systematic Review

**DOI:** 10.7759/cureus.64674

**Published:** 2024-07-16

**Authors:** Alessandro Martinino, Gabriele Campagnoli, Sofia Dallavalle, Allison Soto, Sjaak Pouwels, Frank Smeenk

**Affiliations:** 1 Surgery, Duke University, Durham, USA; 2 General Surgery, International Medical School, Università degli Studi di Milano (La Statale), Milan, ITA; 3 General Surgery, University of Illinois College of Medicine at Chicago, Chicago, USA; 4 Intensive Care Medicine, Elisabeth-TweeSteden Hospital, Tilburg, NLD; 5 School of Health Professions Education (SHE), Maastricht University, Maastricht, NLD

**Keywords:** education, scientific publishing, scholarity integrity, predatory journals, ethics

## Abstract

This systematic review aims to identify the countries most active in combatting predatory journals and their definitions of such practices. It also seeks to assess awareness within academic communities, examine the impact of predatory journals on research quality and integrity, and compile existing policies to mitigate their negative effects and strengthen global scholarly integrity. A systematic search was performed in the PubMed, Scopus, and Embase databases on February 7, 2024, in line with Preferred Reporting Items for Systematic Reviews and Meta-Analyses (PRISMA) guidelines. The focus was solely on identifying studies that examined the unique experiences and interventions associated with predatory journals in distinct national contexts. The analysis included a presentation of quantitative results and a thematic examination of qualitative data. A total of 40 articles covering 19 countries were included. Twenty-four countries (60%) were in Asia, 11 (27.5%) in Africa, two (5%) in Europe, and one (2.5%) each in Australia, North America, and South America. Although not all articles cited Beall's list to identify predatory journals, the thematic analysis showed consistent topics across various definitions and Beall's themes. Our analysis identified factors affecting academic publishing perceptions globally, highlighting publication pressure, predatory practices, and policy impacts on ethics and standards. This systematic review examined the literature on predatory publishing and identified the leading countries in the fight against these predatory publications. This analysis underscores a complex interplay of factors affecting academic publishing globally, from the push towards predatory journals as a response to publishing pressures, to the critical role of government and institutional frameworks.

## Introduction and background

In the realm of academic publishing, predatory journals represent a significant concern, leveraging the open-access model primarily for financial gain while neglecting the principles of transparency and rigorous peer review [1-5]. These practices not only compromise the trustworthiness and validity of the research they publish but also pose a widespread threat to the integrity of scholarly records [6-8]. It is known that the influence of predatory journals does not manifest uniformly across the globe; rather, it varies significantly, shaped by local perceptions and the academic community's exposure to such practices [8]. 

This systematic review is designed to delve into the nuances of these country-specific perceptions and to assess the uneven impact predatory journals have on scholarly integrity. Specifically, this study aims to identify the countries most active in combatting predatory journals and their definitions of such practices. Indeed, acknowledging the variability in the definition of predatory journals worldwide enriches our analysis, allowing us to address the issue with the complexity and depth it demands. It also seeks to assess awareness within academic communities, examine the impact of predatory journals on research quality and integrity, and compile existing policies to propose a unified strategy to mitigate their negative effects and strengthen global scholarly integrity.

This study contributes to the critical discourse on ethical academic publishing and the preservation of research integrity. We aim to enrich the dialogue surrounding ethics in academic publishing and support the development of a scholarly ecosystem that is both informed and robust against the challenges posed by predatory practices [9-12]. Also, by dissecting the landscape through a geographical lens, we intend to uncover distinct patterns and strategies that could inform targeted interventions to mitigate the threat of predatory journals. The significance of understanding these localized dynamics cannot be overstated, as it is crucial for developing nuanced solutions that respect and address the specific needs and challenges faced by the academic communities in different regions.

## Review

Methods

Within the scope of this research, we conducted a systematic review of the literature to pinpoint studies that address the impact and perceptions of predatory journals across different countries. This thorough strategy was designed to deepen our grasp of the topic and offer meaningful contributions to the academic discourse. An AMSTAR 2 (Assessment of Multiple Systematic Reviews-2) checklist is included as supplementary material to facilitate the assessment of the presented systematic review [13,14]. 

Search Strategy

Our research encompassed an exhaustive computerized exploration of the PubMed, Scopus, and Embase databases. The search terms used were "predatory journals", "predatory conferences", and “predatory publishing". Articles were also identified from reference lists of the published articles. The last of these searches were carried out on February 7, 2024.

Inclusion and Exclusion Criteria

For the inclusion criteria of our systematic review, we focused exclusively on selecting studies that delve into experiences and interventions specific to individual countries in the context of predatory journals. This encompassed articles, reviews, case reports, and analyses that present in-depth discussions on the approaches, strategies, and policies implemented within a particular geographical context. This approach ensured that our analysis was focused solely on sources that provided relevant insights and information pertaining to the topic at hand, enhancing the precision and relevance of our research findings. We excluded all non-English language studies and conference abstracts.

Objectives

The primary objective of this study was to identify the countries that have been most active in promoting initiatives against predatory journals and to explore how these regions define predatory publishing practices. 

The secondary objectives were multifaceted. Firstly, we aimed to evaluate the awareness within academic and research communities, reporting the local resources at their disposal to enhance knowledge. Secondly, we intended to explore the repercussions of predatory journals on the quality and integrity of scholarly research at the local level and how these publications undermine the credibility of the academic environments. Lastly, we aimed to compile the array of policies and strategies previously suggested. By aggregating these recommendations, our goal was to present a unified strategy to effectively confront and mitigate the adverse effects of predatory publishing, thereby reinforcing the foundations of scholarly integrity across the global landscape.

Study Selection

**Figure 1:**
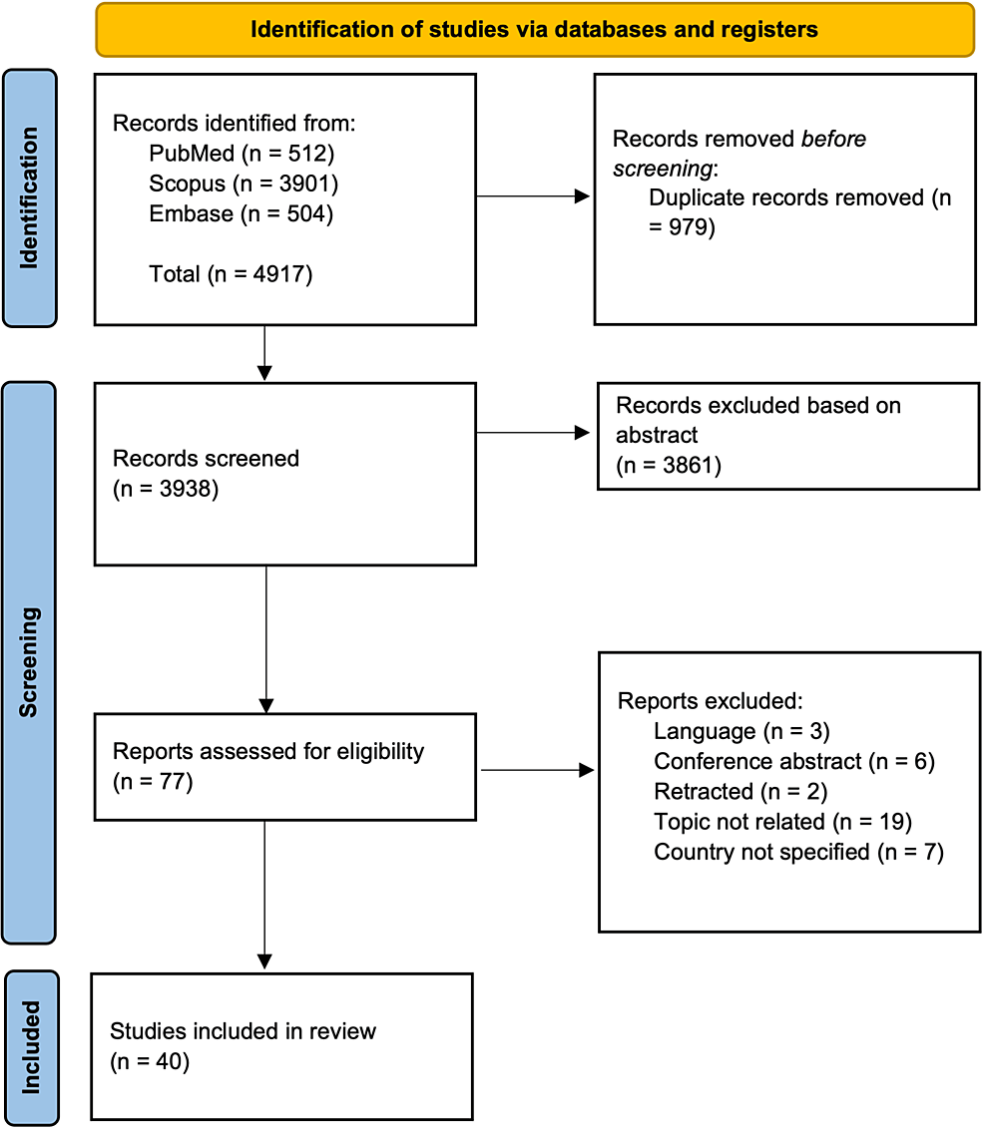
PRISMA Flow Diagram. PRISMA: Preferred Reporting Items for Systematic Reviews and Meta-Analyses

Titles and/or abstracts of studies identified using our search criteria were screened independently by two authors (AM and AS) to identify all studies meeting our inclusion criteria. Any disagreement was resolved through discussion with a third reviewer (SP). A total of 77 studies were assessed for eligibility by reviewing the full text. Figure 1 provides the Preferred Reporting Items for Systematic Reviews and Meta-Analyses (PRISMA) flow chart for this [15]. We did not perform a meta-analysis because of inconsistent reporting of outcome measures and differences in study designs.

Data Extraction

Two authors (SD and GC) reviewed independently the full texts for inclusion and data extraction. A total of 40 studies were deemed eligible for inclusion. AM then reviewed all articles, re-checked data, and collected them using an Excel (Microsoft Corporation, Redmond, Washington, United States) - R (R Foundation for Statistical Computing, Vienna, Austria) sheet. 

Statistical Analysis

A qualitative analysis was conducted using the reflexive thematic analysis technique [16,17]. This technique was used to compare the definitions of predatory journals and to quantify the prevalence of specific terms, contextual nuances, and concepts within selected definitions. The software NVivo (Lumivero LLC, Denver, Colorado, United States) was used to assist and manage the qualitative analysis in this study.

Initially, authors AM and AS independently reviewed and acquainted themselves with the dataset. Subsequently, they employed the reflexive thematic analysis method to code the definitions. The data was approached using an inductive approach, whereby coding and theme development were guided by the content of the responses [18]. The purpose of this approach was to remove any biases or personal interpretations the authors might have had, ensuring that the codes were derived solely from the dataset. The dataset underwent two additional reviews to guarantee coding saturation, meaning no new codes were identified or generated. After the independent examination and coding, the authors convened to discuss their findings, consolidating some codes and identifying patterns within them. Any disagreement was resolved through discussion with a third author (GC).

As the process of reading, deep engagement with the data, and coding unfolded, reflective memos and annotations were produced. From these codes, themes were discerned, evolving as their underlying associations were analyzed and contemplated. Subsequently, these themes underwent a refinement process and were categorized, with a mindful consideration of the reflective memos and annotations that had been compiled earlier. The development of themes was conducted in a semantic manner, focusing on summarizing and reflecting the essence of the dataset’s content.

When referring to Beall's definition of predatory journals, the following applies: “*readily available journals, that accept and are likely to publish all papers submitted to them, as long as the authors are willing to pay the article processing charges (APCs). Usually, the editorial quality of these papers is very poor, and the content is not valid nor validated. In addition, the pedigrees of the journals, and the identity of their proprietors, are often unknown. There is no archiving practice leading to lack of access to their back numbers, and there is doubt about the veracity of their locations*” [19,20]. 

Results

Results of the Search Strategy

Our initial search resulted in 4917 articles. After removing duplicates and title and abstract screening, a total of 77 articles published by February 7, 2024, were identified for full-text assessment. A total of 40 articles were included. Figure 1 highlights our search strategy and PRISMA Flow Diagram. 

In terms of study design, 10 articles were bibliometric analyses, representing 25% of the total, 9 (22.5%) opinion articles, eight (20%) were qualitative research studies, and seven (17.5%) were quantitative research studies. A total of four articles (10%) combined quantitative and qualitative methods and two articles (5%) combined bibliometrics analysis and qualitative research. The study characteristics are shown in Table 1. 

**Table 1:** Study characteristics.

Author	Year	Country	Methodology	Definition of Predatory Journals	Goal of the Study
Omobowale et al. [21]	2013	Nigeria	Qualitative research (interview-based)	Open access international journals with poor review record and pedigree, lacking rigorous academic quality and review system, largely interested in profit making, with numerous typographical errors.	Investigating predatory journals in Nigeria as a dimension of peripheral scholarship and academic dependency.
Nwagwu et al. [22]	2015	Nigeria	Bibliometric analysis	Beall’s definition	Investigating Nigerian publishers in Beall’s list (2012) if they conform to basic publishing practice.
Shuva et al. [23]	2016	Bangladesh	Quantitative research (survey-based)	Journals that charge authors for their publication without giving quality peer-review, copy-editing, and indexing services.	Understanding Bangladeshi faculty members’ awareness, perceptions, and use of open access journals.
Seethapathy et al. [24]	2016	India	Quantitative research (survey-based)	Beall’s definition. Journals with misleading titles that usually reach out researchers with spam emails.	Estimating which category of educational and research institutes predominately publishes in predatory open access journals in India and understanding whether academicians in India are aware of predatory journals.
Erfanmanesh et al. [25]	2017	Iran	Bibliometric analysis	Beall’s definition. Journals utilizing bogus impact factors, appointing well-known academics as editorial board members without their consent, bombarding researchers with spam emails.	Raising the awareness of scholars, especially in Iran, about predatory publishing and its negative consequences.
Pal et al. [26]	2017	India	Opinion article	Beall’s definition.	Highlighting that India being among the largest contributors to manuscripts submitted to predatory journals is surprising, being India a relatively cash-deprived country.
Chilimo et al. [27]	2017	Kenya	Quantitative and qualitative research (survey-based)	Low-quality publishers funded by article processing charges.	Measuring Kenyan researchers’ awareness and general perceptions of OA, past publication in OA journals, the status of OA publications for tenure and promotion at their institution, and their participation in the author-pay model of OA.
Mouton et al. [28]	2017	South Africa	Bibliometric analysis	Beall’s definition. Journals with incomplete or inaccurate information on the members of the editorial board, fake claims about indexing of the impact factor.	Estimating the extent of predatory publishing amongst South African academics.
Patwardhan [29]	2017	India	Bibliometric analysis	Beall’s definition.	Explaining the flows in India that make publishing in predatory journals possible.
Memon [30]	2017	Pakistan	Bibliometric analysis	Journals publishing papers for money and abusing the open access model.	Suggesting how Pakistani universities might avoid predatory journals and improve the quality of research.
Ifijeh [31]	2017	Nigeria	Opinion article	Beall’s definition. Journals that ensure timely publication and open access for easy citations.	Examining crucial issues and implications of predatory publishing among Nigerian academic librarians.
Priyadarshini [32]	2018	India	Opinion article	Beall’s definition	Creating awareness in predatory journals targeting Indian universities.
Madhan et al. [33]	2018	India	Qualitative research	Journals that seduce researchers with offers of membership in editorial boards.	Analyzing misuse of metrics and poor research evaluation practices in India.
Ajuwon et al. [34]	2018	Nigeria	Opinion article	Beall’s definition. Journals whose primary interest is profit and not the promotion of access to scientific knowledge.	Summarizing the published literature on predatory open access publishing, discussing its potential impact on scholarship in Nigeria and offering suggestions to address the problem.
Patwardhan [35]	2019	India	Opinion article	Journals that severely compromise scientific scholarship, collect fees from university students and other authors willing to publish for them, but do not perform peer review or other promised services.	Explaining how Indian universities are reacting to predatory journals affecting the Indian academic community.
Kassian et al. [36]	2019	Russia	Bibliometric analysis	Journals recurring to fake reviewing and auto-reviewing, text loaning without citing, rapid publication by paying APCs, journal self-citation higher than 50%.	Evaluating the journals of the Russian Science Citation Index.
Grgić et al. [37]	2019	Croatia	Quantitative research (survey-based)	Low-quality journals, using article processing charges, lacking peer review, and adopting unethical practices.	Exploring researchers’ and librarians’ awareness of predatory journals, using the example of Croatia.
Atiso et a. [38]	2019	Ghana	Qualitative research (interview-based)	Beall’s definition. Journals emulating highly ranked journals.	Investigating the understanding that research scientists in Ghana have about predatory journals.
Pisár et al. [39]	2019	Czech Republic and Slovakia	Bibliometric analysis and qualitative research (interview-based)	Beall’s definition.	Analyzing the effects of performance funding in the specific conditions of Czech and Slovak institutions of higher education.
Misra et al. [40]	2019	India	Qualitative research	Beall’s definition.	Discussing emergence of OA publishing, initiatives for OA publishing and the role of publishing in academic promotions in India.
Joubert et al. [41]	2019	South Africa	Opinion article	Beall’s definition.	Describing the experience with South African Family Practice regarding response types and times and, to provide some context, compare these with other South African (SA)-based journals.
Sotomayor-Beltran [42]	2019	Peru	Qualitative research (interview-based)	Journals with the purpose to make profit rather than promoting good science.	Presenting the research environment in Peru by analyzing the current situation in Peruvian universities from the professors' and lecturers' perspective.
Tella [43]	2020	Nigeria	Qualitative research (interview-based)	Journals publishing on a open access platform, collecting APC as a condition of publication, and that do not control the quality of submitted material with peer review.	Finding out whether Nigerian academics acknowledge predatory journals and whether they are patronizing them, identifying the characteristics of predatory in Nigerian academics, and suggesting measures that can be taken to stop the patronage of predatory publishers by Nigerian academics.
Downes [44]	2020	Australia	Bibliometric analysis	Beall’s definition.	Determining the nature of their ethical or unethical practices and the extent to which Australian academics were included on the editorial boards of their journal.
Owolabi et al. [45]	2020	Nigeria	Quantitative and qualitative research (survey and interview based)	Journals with only financial aims, with false indexing metrics, sending unsolicited mails requesting for submission of papers.	Investigating the awareness and knowledge of predatory journals among academic librarians in five selected Nigerian universities.
Singh [46]	2020	India	Quantitative and qualitative research	Journals publish research of academicians without following any publishing guidelines and seek financial profits through APC.	Presenting an overview of quantity and quality of medical research in India, giving insight into quantity and quality of medial research in the country.
Babb [47]	2021	Canada	Bibliometric analysis	Journals that prioritize self-interest at the expense of scholarship and that are characterized by false or misleading information, deviation from best editorial and publication practices, lack of transparency, and/or the use of aggressive and indiscriminate solicitation practices.	Exploring publishing trends in predatory journals by authors affiliated with Canadian universities and the differences in predatory journal publications between Canada’s research-intensive U15 universities and other, non-U15 universities.
Patwardhan et al. [48]	2021	India	Opinion article	Beall’s definition.	Discussing the UGC-CARE initiative, its structure, objectives and specifically, “UGC-CARE Reference List of Quality Journals” (UGC-CARE list) and finally, the challenges it faces.
Eshchanov et al. [49]	2021	Uzbekistan	Bibliometric analysis	Journals recurring to aggressive marketing through emails, the imitation of top journals’ names, little geographical diversity among the editorial board and authors, and quick acceptance of articles.	Reviewing the principles of implementation of the “publish or perish policy” in Uzbekistan with an overarching aim of detecting the factors that are pushing more and more scholars to publish the results of their studies in predatory journals.
Rim [50]	2021	South Korea	Systematic review	Journal that primarily pursues profits without applying the methodology to ensure the academic quality.	Examining recent trends in academic from international and South Korean perspectives, and the significance of open-access publishing and recent changes.
Wang et al. [51]	2021	China	Quantitative research (survey-based)	Beall’s definition.	Investigating the attitudes of Chinese PhD students toward predatory journals.
Hegde et al. [52]	2021	India	Opinion article	Journals born from a vicious cycle fueled by systemic failure.	Demonstrating that an urgent change to the system is required, by the regulating bodies clearly defining best practices in publishing, leaving no scope for predatory journals to mislead authors.
Koçak [53]	2022	Türkiye	Qualitative research	Beall’s definition.	Giving a perspective about decisions of the Turkish Council of Higher Education (TCHE) and the imperative need to find other strategies to regulate the issue of predatory journals in Türkiye.
Kim et al. [54]	2023	South Korea	Qualitative research (interview-based)	Corrupted open access journals.	Diagnosing challenges and strengths of OA journal publishing practices, identifying the areas that require efforts for enhancement, and providing a roadmap to progress in improving OA journal publishing practices.
El Bairi et al. [55]	2023	Morocco	Quantitative research (survey-based)	Fraudulent journals characterized by well-known hallmarks such as fake metrics, unsolicited mail invitations, inappropriate websites, unqualified or fabricated editorial boards, guaranteed manuscript acceptance, low article-processing charges, and the nonexistence of peer-review process.	Evaluating the use of training based on distance education, surveillance of researchers by their mentors and affiliations, and social networks to increase awareness on predatory publishing.
Balakumar et al. [56]	2023	India	Opinion article	Journals that can seriously undermine research integrity.	Training initiative has been developed and could warn the researchers about the dangerous effects undermining the predatory journals prevalence in India.
Trubnikov et al. [57]	2023	Russia	Quantitative research	Beall’s definition. Journals that promote dishonest behavior of academics and publishers, also creating an environment favorable for corruption, leading to the overall degradation of the participating academic organizations.	Analyzing the main underlying reasons for the explicit predatory choice by Russian universities.
Wang et al. [58]	20233	China	Quantitative research (survey-based)	Journals that prioritize self-interest at the expense of scholarship, characterized by false or misleading information, deviation from best editorial and publication practices, a lack of transparency, and/or the use of aggressive and indiscriminate solicitation practices.	Describing Chinese scholars of the biomedical field contribution to predatory journals.
Dora et al. [59]	2023	India	Bibliometric analysis	Beall’s definition.	Exploring predatory journals in LIS subject published from India, including an analysis of the affiliation status of the authors publishing in those predatory journals, frequency of returning authors, and country-wise distribution of authors.
Sharma et al. [60]	2023	India	Qualitative research	Beall’s definition.	Determining the factors behind the expansion and promotion of predatory journals in India.

Primary Objective 

The main objective of this review is to pinpoint the nations that are leading the charge in initiatives against predatory journals and to delve into the ways these regions define the practices of predatory publishing. Regarding the geographic distribution, there are 24 countries in Asia (60%) included in this review, with the breakdown being 13 in India, two in Russia, two in South Korea, two in China, and one each in Uzbekistan, Bangladesh, Iran, Pakistan, and Türkiye [23-26,29,30,32,33,35,36,40,46,48-54,56-60]. Africa is the setting for 11 (27.5%) studies, including six in Nigeria, two in South Africa, and one each in Kenya, Ghana, and Morocco [21,22,27,28,31,34,38,41,43,45,55]. Europe has two (5%) studies, split between Croatia and a combined entry for the Czech Republic and Slovakia [37,39]. Additionally, there's one study based in Australia (Australia) (2.5%), one in North America (Canada) (2.5%), and one in South America (2.5%), specifically in Peru [42,44,47]. A total of 19 countries were analyzed. 

**Figure 2:**
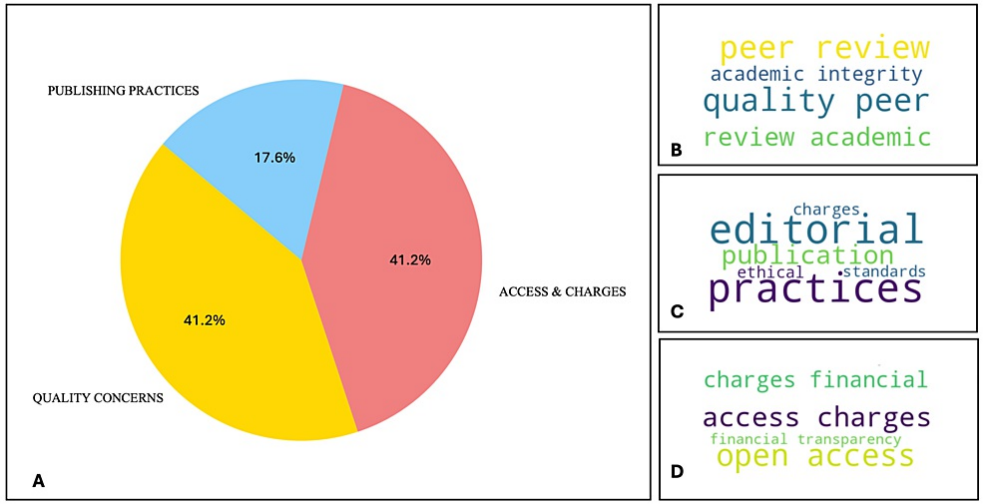
Distribution of themes in definitions of predatory publishing (A); Word cloud for Quality Concerns (B); Word cloud for Publishing Practices (C); Word cloud for Access and Charges (D).

Table 1 outlines the definition of predatory journals as defined by each article. Of these, 20 articles refer to Beall's list, while another 20 provide their own definitions. A thematic analysis was conducted on the latter 20 definitions, with details provided in Figure 2, and it revealed three main themes. First, "Quality Concerns", which emphasize issues surrounding academic integrity, the peer review process, and the general quality of publications (Figure 2B). Second, "Publishing Practices", which delves into ethical dilemmas, the adherence to editorial standards, and the transparency of the publication process (Figure 2C). Lastly, "Access and Charges", which focuses on the economic models employed by publishers, specifically the openness and fairness of fees charged to authors for open-access publishing (Figure 2D).

Even though not all articles directly referenced Beall's list, the thematic analysis revealed similar and congruent topics between the various definitions and the themes identified within Beall's list. This consistency underscores a shared understanding of the core issues surrounding predatory journals across different country perspectives. Table 2 details thematic occurrences across all continents, including in this analysis both the independent definitions and those referencing Beall's list, for a total of 40 definitions, thereby offering a comprehensive global overview. In Africa, there are 11 definitions, with a significant emphasis on both Quality Concerns and Access and Charges, evidenced by nine and 11 mentions respectively. Publishing Practices are also notable, with seven instances. Asia, contributing the most definitions at 24, shows a balanced concern across all three themes, Quality Concerns, Publishing Practices, and Access and Charges, each mentioned 15 times. This suggests a comprehensive concern across various aspects of predatory publishing. Australia, with only one definition, and Europe, with two, show concerns across all themes, indicating a universal recognition of these issues in predatory publishing definitions, despite the low number of contributions. North America has a single definition, emphasizing on Publishing Practices with no mention of Quality Concerns, highlighting a possible regional difference in focus. This analysis underscores the varied emphasis on predatory publishing themes across continents, reflecting potentially diverse regional experiences and concerns within the academic community.

**Table 2:** Predatory publishing definition: thematic occurrences across all continents.

Continent	Total number of definitions	Quality Concerns	Publishing Practices	Access and Charges
Africa	11	9	7	11
Asia	24	15	15	15
Australia	1	1	1	1
Europe	2	2	2	2
North America	1	0	1	0

Secondary Objectives 

Our analysis reveals significant insights into the factors influencing the perception of academic publishing across various countries, highlighting issues such as the pressure to publish, predatory publishing practices, and the impact of government and institutional policies on publication ethics and standards. Predatory practices are a notable concern, with countries like Nigeria, Canada, China, Korea, Morocco, Peru, Russia, South Korea, and Uzbekistan specifically mentioning challenges related to the rise of predatory journals. In India, the pressure to publish is particularly emphasized, pointing to a culture where academic success is tightly linked to publication output [40,46,52]. This pressure is also acknowledged in China, indicating a widespread challenge across different academic landscapes [51].

In Nigeria and Bangladesh, a notable gap in awareness is evident, with nearly 50% of faculty members at the University of Dhaka being unaware of the author-pay model, underscoring a broader trend of insufficient knowledge about predatory journals [22,23,45]. This is mirrored in India, where approximately 57% of researchers remain oblivious to these malpractices [24].

Meanwhile, Croatia faces unique economic and access challenges, struggling with the high costs of reputable publications and a lack of awareness around open-access alternatives, which could mitigate some of these barriers [37]. This situation underscores the disparities in access to quality publishing platforms, further exacerbated by economic factors. Socioeconomic factors play a critical role also in India and unspecified African nations, where researchers, driven by the need to disseminate their findings, are more inclined to engage with predatory journals, highlighting the impact of economic and geopolitical challenges on academic integrity [29,31,33-35,38,48,52]. 

The theme of integrity was also highlighted in most of the papers included. A particularly egregious practice involves predatory journals listing legitimate researchers as editorial board members without their consent, a tactic noted in Iran, which further jeopardizes scholarly integrity by lending unearned credibility to these predatory outlets [25]. Also, Wang et al. conducted the first study to survey Chinese authors who published in predatory journals within the biomedical field. The study describes a significant incident in 2021, where the European Review for Medical and Pharmacological Sciences retracted 199 papers authored by Chinese academics due to academic misconduct. This incident underlines the concern that the prevalence of submissions to predatory journals by Chinese researchers could tarnish their global reputation and hinder academic exchanges [58].

Countries such as Bangladesh, China, Croatia, the Czech Republic, Slovakia, Ghana, India, Iran, Morocco, Nigeria, South Africa, and Turkey have underscored the critical importance of both institutional and individual accountability [22,25,31,33,35,37-40,43,46,48,51,53,55,58,59]. Simultaneously, countries like Australia and Russia are making strides in enhancing the quality of scientific research and its dissemination by reinforcing the principles of publication ethics [44,57]. India demonstrates a proactive stance against predatory practices, evidenced by initiatives like the UGC-approved journal list aimed at guiding academics toward reputable publishing platforms [48]. Also, Nigeria stands out for its institutional vigilance, with promotion committees increasingly rejecting publications in predatory journals, a measure not as pronounced in Pakistan, which faces a gap in comprehensive strategies to combat such predatory practices [30]. Similarly, the Czech Republic and Slovakia present a case where government and institutional policies have inadvertently encouraged practices that favor quantity over quality, highlighting the need for a balanced approach that prioritizes ethical standards and research integrity [39]. 

Discussion

In the academic world, the integrity of scholarly publishing serves as a cornerstone for the advancement of knowledge, ensuring that research is conducted, reviewed, and disseminated under rigorous ethical standards. This integrity is vital for the credibility of scientific discourse, the progression of various fields of study, and the preservation of trust between researchers, institutions, and the public. However, the rise of predatory publishing practices poses a significant threat to this foundational principle, challenging the very essence of scholarly communication. Our systematic review was designed with two primary objectives: initially, to pinpoint the countries leading the efforts against predatory journals and examine their definitions of predatory publishing practices, and subsequently, to evaluate the impact of predatory journals on academic and research communities, detailing the resources available for improving awareness and compiling a list of previously suggested strategies.

A collection of 40 articles from 19 distinct countries was included. Despite not all articles making direct references to Beall's list's definition of predatory journals, the thematic analysis pointed to a notable similarity and alignment in topics across the various definitions and those identified in Beall's list. Such alignment signifies a mutual recognition of the essential problems associated with predatory journals, reflecting a consensual viewpoint across diverse international perspectives. The analysis revealed intricate dynamics shaped by awareness, socioeconomic factors, institutional policies, and ethical considerations. Across the board, there's a clear need for enhanced education and guidelines to navigate the complex landscape of scholarly publishing, underscoring a universal challenge in raising awareness and fostering ethical publishing practices. This panorama also hints at a gap between individual researchers' awareness of predatory journals and their willingness to acknowledge personal participation, suggesting a nuanced battleground of personal ethics and responsibility within the academic community [43].

The impact is especially pronounced in developing countries, where the infiltration of low-quality publishers can severely damage the reputation of local researchers and their contributions to scientific literature [61]. This situation is exacerbated by a lack of effective mechanisms to combat fraudulent publishers and insufficient guidance for researchers on reputable publishing avenues. For instance, Kenya faces significant risks from such practices, which threaten to damage the reputation of researchers, undermine research output, and further marginalize its academic community [27]. Moreover, predatory publishing has catalyzed the formation of academic networks that thrive on reciprocal "gifts" and privileges, rewarding participants adept in manipulative publishing practices. This fosters an environment where scholarly integrity is compromised in favor of visibility and perceived academic success. In our systematic review, we discovered that articles and initiatives based in North America and Europe were surprisingly scarce. Nonetheless, this phenomenon isn't confined solely to developing nations. A particular study revealed that over half of the corresponding authors hailed from countries classified as high- and upper-middle-income by the World Bank. Within their dataset, the United States contributed more articles than any other country, with the exception of India. Additionally, it was observed that among the 17% of articles that disclosed their funding sources, the most commonly cited sponsor was the United States National Institutes of Health (NIH) [62,63].

Examining the different global strategies to combat this challenge, we observed a diverse array of recommendations and solutions tailored to the unique contexts of different countries. For instance, authors from Bangladesh present a structured approach by establishing a committee of deans across faculties [23]. This method highlights the importance of institutional engagement, showing how structured, collective action within educational institutions can act as a powerful mechanism for change. Erfanmanesh et al., from Iran, advocate for a proactive personal stance against questionable journals, demonstrating a keen awareness of the pitfalls in academic publishing [25]. They promote a culture of vigilance and informed decision-making, emphasizing the critical role of thinking skills. Despite the variety of solutions proposed, the principles of collaboration, awareness, and education stand as the cornerstone in devising effective responses to this complex issue.

Predatory publishing reflects a broader global trend toward the commodification of academic publishing, where the integrity of the publication process is often compromised [64]. The commodification of academic publishing denotes a significant shift towards viewing academic outputs as market commodities, thereby subjecting them to the dynamics of market economics. This transformation implies that the dissemination of scholarly work is increasingly influenced by profit motives and market demands. Such a trend manifests through practices like prioritizing content that promises higher financial returns and valuing publications based on metrics that may not accurately represent the research's quality or societal impact. This commodification raises pressing concerns about the accessibility and integrity of scholarly communication, as well as its inclusiveness, potentially sidelining valuable research that does not conform to market-driven criteria.

## Conclusions

Our systematic review, which included 40 studies, aimed to thoroughly examine the literature on predatory publishing and identify the leading countries in the fight against these predatory publications. This analysis suggests a general agreement on the definition of predatory journals across a variety of international perspectives and underscores a complex interplay of factors affecting academic publishing, from the push towards predatory journals as a response to publishing pressures, to the critical role of government and institutional frameworks in shaping the academic publication landscape. Interestingly, most efforts were concentrated in Asia and Africa, while North America and Europe had notably fewer articles and initiatives. The insights gathered from this analysis illuminate the varied challenges faced by researchers worldwide, calling for a concerted effort to address these issues and promote a more ethical, accessible, and equitable academic publishing environment.
